# Risk-aware survival time prediction from whole slide pathological images

**DOI:** 10.1038/s41598-022-26096-z

**Published:** 2022-12-19

**Authors:** Zhixin Xu, Seohoon Lim, Hong-Kyu Shin, Kwang-Hyun Uhm, Yucheng Lu, Seung-Won Jung, Sung-Jea Ko

**Affiliations:** 1 Department of Electrical Engineering, Korea University, Seongbuk-gu, Seoul, 02841 South Korea; 2 Education and Research Center for Socialware IT, Korea University, Seoul, 02841 South Korea

**Keywords:** Image processing, Machine learning

## Abstract

Deep-learning-based survival prediction can assist doctors by providing additional information for diagnosis by estimating the risk or time of death. The former focuses on ranking deaths among patients based on the Cox model, whereas the latter directly predicts the survival time of each patient. However, it is observed that survival time prediction for the patients, particularly with close observation times, possibly has incorrect orders, leading to low prediction accuracy. Therefore, in this paper, we present a whole slide image (WSI)-based survival time prediction method that takes advantage of both the risk as well as time prediction. Specifically, we propose to combine these two approaches by extracting the risk prediction features and using them as guides for the survival time prediction. Considering the high resolution of WSIs, we extract tumor patches from WSIs using a pre-trained tumor classifier and apply the graph convolutional network to aggregate information across these patches effectively. Extensive experiments demonstrate that the proposed method significantly improves the time prediction accuracy when compared with direct prediction of the survival times without guidance and outperforms existing methods.

## Introduction

In the medical analysis domain, survival analysis aims to predict the time of death, cardiac arrest, or occurrence of a specific disease^1^. Accurate survival prediction can help doctors make correct diagnoses with fewer mistakes, thereby improving the treatment quality and quality of life among patients. Based on advances in medical imaging techniques, doctors can take advantage of visual and non-visual features such as genomic composition, clinical information, and treatment variables^2–11^, to determine the best course of treatment. In particular, pathological images available as whole slide images (WSIs) provide abundant information, particularly helpful for cancer diagnosis^12^. Especially, tumor tissue can be identified from WSIs, which is crucial for cancer diagnosis for pathologists^13^.

Over the past decade, deep learning has remained at the center of computer vision research based on its ability to train complex models. Consequently, deep-learning-based WSI analysis has emerged as an active research field, providing a new approach to medical diagnosis and treatment. Typically, a single WSI consists of billions of pixels containing rich cellular-level visual information^14^. However, difficulties arise when processing ultra-high-resolution images, particularly when a neural network is used. As a result, WSIs are typically cropped into small-size patches, and patch-level predictions are performed^14,15^. Studies on the aggregation of patch-level predictions have also been conducted to enable patient-level predictions^16^.

Recently, several deep-learning-based methods have been introduced to predict the survival risk from pathological images^5,14,16–20^. Based on the Cox model^21^, these methods can predict the death occurrence order of patients. However, direct survival time prediction for each patient is only feasible with further estimation of a baseline hazard function. Because the baseline hazard function is an unspecified, nonnegative function^22^, a variety of methods have been proposed to predict it^6,23,24^, and the results vary depending on different estimation strategies, leading to sub-optimal survival time prediction performance. One recent study attempted to predict survival time directly based on the regions of interest (ROIs) in WSIs^15^.

We observed that most existing methods either focused on ranking the death occurrences of patients or predicting survival times, which does not provide sufficient information in practical diagnosis since the prediction of the death order among patients and the survival time for each patient are both essential. An accurate time prediction provides doctors with a quantitative result they can rely on to develop more reasonable treatment plans. Additionally, the order of death provides a reference for doctors to evaluate the progression of the disease (e.g., how critical is the situation of patient A compared with that of patient B) because survival prediction is basically a regression problem^25^. Therefore, it is important to predict survival times accurately and in the correct sequence. To this end, we propose to take advantage of both risk prediction and time prediction by allowing the estimated survival risk features to guide death ranking, thereby enabling the prediction of survival times with higher accuracy in the correct sequence. To better aggregate patch-level features to make WSI-level predictions, we construct a graph by connecting patches in each WSI. To eliminate as much interference (e.g., normal tissue regions which do not contribute to the diagnosis) as possible without extra manual annotation, instead of using all tissue patches within WSIs or randomly sampling patches, we select patches detected as cancer by a pre-trained patch-level binary classifier. Subsequently, a graph convolutional network (GCN) is used to aggregate information across patches effectively.

Our main contributions are threefold. First, we propose a two-branch GCN-based model that integrates risk and time prediction features to make more accurate predictions. To the best of our knowledge, this is the first work to use survival risk prediction as guidance to predict exact survival times of patients from WSIs. Second, we design our framework to predict survival times directly by effectively aggregating patch-level information through GCN. A distinct difference compared to existing survival time prediction works is the prediction is made on WSI-level while manual annotation of ROIs are not required. Third, through extensive experiments, we show that our model achieved superior performance and generalization ability on several publicly available datasets in terms of the mean absolute error for survival time prediction.

## Related work

Automatic survival prediction from pathological images is of significant help to doctors in diagnosis. Based on recent advances in deep learning, learning-based methods are receiving significant attention. Here, we review literature in this field from three perspectives: (1) Prediction level (i.e. patch-level vs. WSI-level), (2) network (i.e. CNN vs. GCN), and (3) output (i.e. risk vs. survival time).

First, due to the extremely large image size of WSIs, most neural networks cannot handle WSIs directly. Therefore, one research direction is to explore the potential of neural networks for survival prediction using small-size patches^14,15,26^. Because multiple patches can be extracted from a single WSI, a training dataset consisting of a sufficient number of patches can be easily obtained, facilitating the research on survival prediction. To make it feasible for survival prediction using a single patch, Zhu et al.^14^ manually acquired the ROI of size $1024\times 1024$ for each WSI and performed a single-patch-based prediction using a deep convolutional neural network (CNN). Although promising results have been reported, such approaches require a significant amount of time and effort from experienced pathologists. Therefore, an alternative approach is to obtain and aggregate patch-level predictions from multiple patches in a WSI. For example, Zhu et al.^16^ clustered multiple patches according to their phenotypes and selected distinctive ones for further aggregation to obtain a WSI-level prediction. Besides, there are also approaches to aggregate the patch-level features so that the prediction is directly made on WSI-level or patient-level^14,17,19,27–31^. Yao et al.^17^ improved patch-level prediction and aggregation by adopting the multiple instance learning framework. Chen et al.^28^ formulated WSIs as a graph-based data structure in the Euclidean space similar to a point cloud such that the node features can be hierarchically aggregated from local to global structures.

Second, network architecture plays an essential role in survival prediction. Motivated by the success of CNNs for general computer vision tasks, early studies on survival prediction are only based on CNNs. Many widely-used CNN architectures, such as VGG^32^, ResNet^33^, and U-Net^34^ have been adopted as baseline network architectures for feature extraction or aggregation of survival prediction^15,29,35^. Chang et al.^29^ proposed to extract patch features using a pre-trained ResNet-50^33^, then construct the feature map from the patch features according to the location of the image patches in the WSI, and apply convolution operation to the generated feature map to predict the survival risk. However, for aggregating patch-level features or predictions to make WSI-level predictions, CNNs are only applicable to regularly structured patches such as tiles^19^. In contrast, GCN^36^ can operate on any graph structure defined from the patches in a WSI and effectively aggregate information across the vertices via graph convolution; thus, it is an alternative design of the CNNs. Li et al.^19^ selected random patches from a WSI and connected them according to Euclidean distances. A graph attention mechanism was applied to aggregate information from important patches among randomly chosen patches. Wang et al.^27^ sampled cancerous patches from a WSI using a patch-level CNN and applied nuclei instance segmentation to each sampled patch to help the survival prediction model learn the hierarchical graph representations.

Third, the type of survival prediction output also needs consideration in clinical practice. Cox-model-based methods have been extensively studied and have achieved outstanding performance in predicting the survival risk of patients^14,16–19,27–29^. However, predicted risk values can only be used to compare the death rankings of patients, which is insufficient for estimating the survival time of each patient directly. Although further estimation of the baseline hazard function can enable survival time prediction, the baseline hazard function is an unspecified function^22^, leading to a variety of ways to predict it^6,23,24^. Meanwhile, Xiao et al.^15^ formulated the patch-level direct survival time prediction as an ordinal regression problem and introduced a censoring-aware loss to deal with the censored data. However, survival time prediction is still conducted at the patch-level, which requires the manual selection of patches.

In summary, WSI-level prediction is more desirable than patch-level prediction for practical diagnosis because of its ability to capture both local and global information without manual ROI annotation. Additionally, to aggregate information across different numbers and positions of patches effectively, GCN is a preferable choice for backbone architecture design. Finally, compared to risk prediction, direct time prediction is more straightforward and helpful for diagnosis. Based on these reasons, we propose to directly predict survival time from WSIs using the GCN backbone.

**Table 1:** Description of the datasets used in our experiments.

Dataset	BLCA			BRCA			GBM		
	Training	Validation	Test	Training	Validation	Test	Training	Validation	Test
#Patients	138	83	119	508	205	304	180	49	57
#WSIs	196	84	120	529	227	325	342	148	150

## Results

### Datasets

We used the public cancer datasets provided by The Cancer Genome Atlas (TCGA) as our experimental datasets. Our experiments were conducted on three bladder, breast, and brain cancer subtypes in TCGA: bladder urothelial carcinoma (BLCA), breast invasive carcinoma (BRCA), and glioblastoma multiforme (GBM). Each dataset contained high-resolution WSIs of patients with a wide range of observation times.

We first extracted patches of size $512\times 512$ for each WSI (for the 20$\times$ magnification scale) and then applied our pre-trained tumor classifier to select tumor patches. We then excluded WSIs that did not contain any patches classified as tumors. Additionally, WSIs with exceptionally large observation times (more than 2000 days for BLCA and GBM or 4000 days for BRCA) were removed, resulting in the three datasets summarized in Table 1. Each dataset was divided into training $(70\%)$ and test $(30\%)$ sets, and we further split $30\%$ of samples in the training set as the validation set. These datasets were used for both the RP-GCN and TP-GCN.

### Implementation details

For graph construction, the node features extracted by ResNet-18 were of size $512\times 1$. The number of neighbors *k* was set to 8, and the distance threshold *d* was set to 2560 pixels at the 20$\times$ magnification scale, which is the total length of five patches. The pooling ratios *r* for the three GCN blocks were set to 0.6, 0.6, and 0.5, respectively.

We trained the RP-GCN for 300 epochs using the Adam optimizer^37^ and set the initial learning rate to 1*e*−4, which was halved after 10, 30, and 50 epochs. For the training of the TP-GCN, we quantized the observation time into several intervals such that each interval contained the same number of training samples^15^. This partitioning led to balanced training samples for the *N* classifiers, where the number of training samples in each interval was seven in our experiments. The initial learning rate of TP-GCN was set to 5*e*−4 while the other settings were the same as those for RP-GCN. We adopted ReLu and tanh as the activation functions $\sigma$ for RP-GCN and TP-GCN, respectively.

### Performance evaluation

Because the proposed model consists of RP-GCN and TP-GCN, we first evaluated the performance of RP-GCN to confirm that the predicted survival risk could be beneficial for the subsequent survival time prediction task. We then evaluated the performance of TP-GCN to verify the effectiveness of the proposed model. Several ablation studies were also conducted to demonstrate the necessity of each component of the proposed model. All experiments on the proposed and compared methods were conducted using the same datasets listed in Table 1.

#### Survival risk prediction

We used the C-index^38^ as our evaluation metric. The C-index evaluates how well the death ranking among patients is organized, and is defined as follows:

1 $$\begin{aligned} C_r=\frac{1}{K}\sum _{i=1}^{P}\delta _{i}\sum _{j:\tau _{i}<\tau _{j}}{\mathbb {I}}_{R_{i}>R_{j}}, \end{aligned}$$

where $C_r$ represents the C-index of risk prediction, *K* indicates the total number of comparable pairs, and *P* is the total number of patients. ${\mathbb {I}}_{a}$ is the indicator function, i.e., ${\mathbb {I}}_{a}=1$ if *a* is true, and ${\mathbb {I}}_{a}=0$ otherwise. During the training stage, each WSI was considered an individual data sample. During the validation and testing stages, if there were several WSIs for one patient, we considered the average value of the WSI-level predictions as the patient-level prediction result. The test results are listed in the first row of Table 3. The C-indexes of risk prediction for BLCA, BRCA, and GBM were obtained as 0.834, 0.627, and 0.563, respectively.

**Table 2:** Results of the proposed and existing methods. Because DeepAttnMISL^17^, PatchGCN^28^, and DeepGraphSurv^19^ are originally survival risk prediction methods, we used the Python package lifelines^39^ to convert the survival risk into survival time for evaluation.

Method	BLCA				BRCA				GBM			
	MAE		C-index		MAE		C-index		MAE		C-index	
	Expect	Thresh	Risk	Time	Expect	Thresh	Risk	Time	Expect	Thresh	Risk	Time
DeepAttnMISL	386.1*	495.6*	0.526	–	172.6*	181.3*	0.652	–	310.0*	296.4*	0.626	–
PatchGCN	330.7*	304.5*	0.518	–	171.9*	205.1*	0.564	–	314.9*	300.5*	0.529	–
DeepGraphSurv	153.5*	167.1*	0.820	–	191.4*	193.9*	0.618	–	351.5*	337.8*	0.550	–
CDOR	329.6		–	0.550	203.3		–	0.561	320.0		–	0.492
Proposed	123.2		–	0.849	167.5		–	0.605	303.3		–	0.554

The evaluation was conducted on both time accuracy (MAE in days) and ranking accuracy (C-index). *Indicates the survival time results calculated by lifelines.

**Table 3:** Ablation study of the proposed model.

Method	BLCA				BRCA				GBM			
	MAE		C-index		MAE		C-index		MAE		C-index	
			Risk	Time			Risk	Time			Risk	Time
RP-GCN	–		0.834	–	–		0.627	–	–		0.563	–
TP-GCN	182.4		–	0.808	178.8		–	0.565	336.6		–	0.542
Proposed	123.2		–	0.849	167.5		–	0.605	303.3		–	0.554

#### Survival time prediction

For the performance evaluation of TP-GCN, we used the mean absolute error (MAE) between the ground truth and predicted survival time, following^15^. Specifically, for each patient with a censoring status $\delta$, the predicted survival time ${\tilde{\tau }}$, and observation time $\tau$, the MAE can be calculated as follows:

2 $$\begin{aligned} \Delta =\frac{1}{P}\sum _{i=1}^{P}(\tau _{i}-{\tilde{\tau }}_{i}){\mathbb {I}}_{{\tilde{\tau }}_{i}<\tau _{i}}+\delta _{i}({\tilde{\tau }}_{i}-\tau _{i}){\mathbb {I}}_{{\tilde{\tau }}_{i}\ge \tau _{i}}. \end{aligned}$$

We also evaluated the pairwise ranking consistency between the predicted survival times by calculating the C-index as follows:

3 $$\begin{aligned} C_t=\frac{1}{K}\sum _{i=1}^{P}\delta _{i}\sum _{j:\tau _{i}<\tau _{j}}{\mathbb {I}}_{{\tilde{\tau }}_{i}<{\tilde{\tau }}_{j}}, \end{aligned}$$

where $C_t$ represents the C-index of survival time prediction. Similar to the performance evaluation of the RP-GCN, we considered each WSI as an individual during the training stage and averaged the results of several WSIs from the same patient to form our final prediction result during the validation and testing stages.

For the sake of fair performance comparisons, we tested three existing methods that perform survival risk predictions at the WSI-level, which are denoted as DeepAttnMISL^17^, PatchGCN^28^, and DeepGraphSurv^19^, in addition to the patch-level direct survival time prediction model CDOR^15^. We conducted the performance comparisons using the same experimental settings introduced in the corresponding studies and the same datasets used in our experiments. For all compared methods, we selected the model with the best performance on the validation set for evaluating the performance on the test set.

Because the other methods, except for CDOR^15^, can only predict survival risk, we followed the approach in^15^, which converts the survival risks predicted by different methods into time results using the Python package *lifelines*^39^. This package can automatically estimate the baseline hazard function $h_0(\tau )$, and consequently, the hazard function $h(\tau |{\pmb {x}})$ of the Cox model can be calculated with the predicted survival risk. The survival function can then be estimated, and finally, the survival time is calculated using two approaches: (1) taking the expectation of the survival function, termed as “Expect”, and (2) thresholding the survival function, termed as “Thresh.” For CDOR, because the original method required manually selected patches of size $1024\times 1024$, which are not publicly available, we trained another tumor classifier using the Camelyon17 dataset^40^ with the patch size $1024\times 1024$ in the same manner as the patch classifier used to construct a graph structure in our experiments. Then, for each WSI, we selected the patch with the highest tumor probability and obtained the performance from the selected patch. The survival time prediction results using the proposed model and other methods are presented in Table 2.

**Figure 1:**
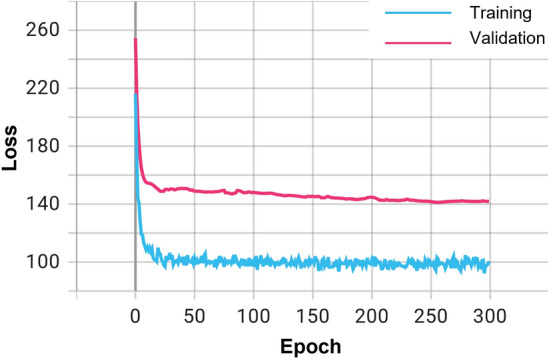
Training and validation loss per epoch on BLCA.

As can be observed, the MAEs of the proposed model were obtained as 123.2, 167.5, and 303.3 days for BLCA, BRCA, and GBM, respectively, outperforming the existing approaches on BLCA and BRCA. Especially for BLCA, the MAE was significantly lower (-30.3 days compared to the second-lowest one obtained by DeepGraphSurv^19^) than the other approaches, while the C-index of the time-prediction results was also higher (+0.029 compared to the second-highest one obtained by DeepGraphSurv^19^) than the other approaches. Figure 1 shows the training and validation loss per epoch on BLCA, demonstrating that no overfitting problem arose. For BRCA, one can see that even though the C-index obtained from DeepAttnMISL was the highest among all approaches, its survival time prediction accuracy is lower than the one obtained from the proposed model. This is because more complicated procedures, such as the estimation of the baseline hazard function, are required to convert the survival risk results into survival time results. As for GBM, the result was slightly worse than DeepAttnMISL and PatchGCN; we considered this might be due to the significant difference between the number of patients and WSIs in GBM (57 and 150). The two risk-prediction approaches both adopted early fusion strategies to aggregate the information of multiple WSIs from the same patient, and consequently, the training and prediction were both performed directly on the patient-level. However, the proposed model was trained on the WSI-level, and the final patient-level results were obtained by averaging the WSI-level results of one patient.

#### Ablation study

We conducted an ablation study to verify the effectiveness of the risk feature ${\pmb {h}}_{r}$. To this end, we directly applied ordinal regression to the time feature ${\pmb {h}}_{t}$ extracted by the TP-GCN and calculated the MAE and C-index of the obtained results. We also reported the C-index of the survival risk prediction results. As shown in Table 3, the C-index obtained from the results using the time feature alone for BLCA, BRCA, and GMB was 0.808, 0.565, and 0.542, respectively, indicating that the survival ranking between patients was not sufficiently handled. When the risk feature was involved, further ranking information could be provided to guide the time prediction, leading to a higher C-index. Meanwhile, the MAEs for the TP-GCN were obtained as 182.4, 178.7, and 336.6 for the three datasets, respectively. However, for the proposed model, because the ranking was also considered during time-prediction training, the MAEs of the TP-GCN were reduced by 59.2, 11.2, and 33.3 days for the three datasets, respectively.

**Figure 2:**
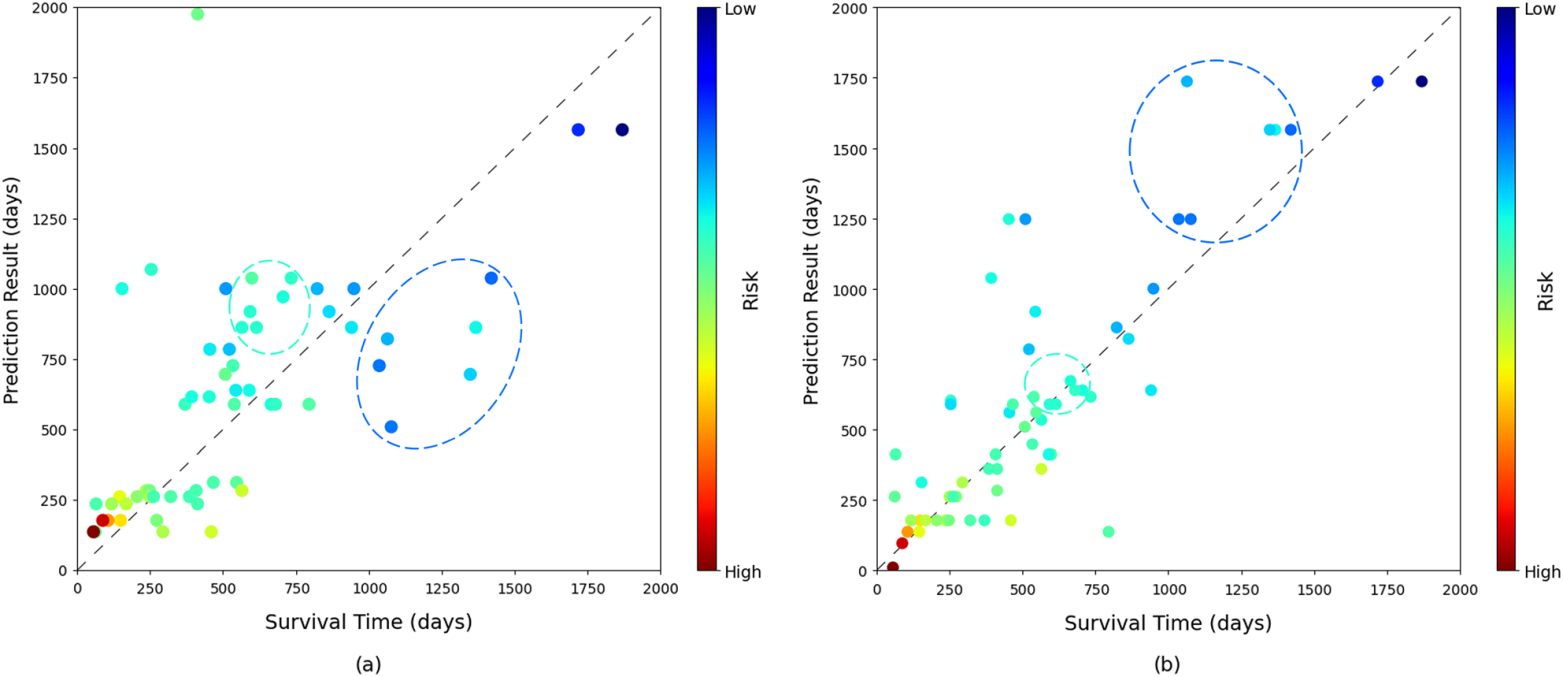
Visualization of the results on the uncensored data obtained using (**a**) the time feature only and (**b**) the risk-time feature. The color represents the predicted risk obtained by RP-GCN for each patient.

Figure 2 visualizes the results on the BLCA test set. Ideally, the closer the point to the dotted diagonal line, the better the performance. Because any points above the line are all correct predictions for the censored data, we excluded the censored data for this figure to better understand the results. When only the time feature ${\pmb {h}}_{t}$ was used, the results were close to the ground truth overall. However, as shown in Fig. 2a, the time prediction results inside the cyan circle are very close to the ones inside the blue circle. In other words, although the prediction results are reasonably close to the ground truth, their death rankings are inaccurate. Notice the color of these points, i.e., the predicted risk. The predicted risks of the data inside the blue circle are lower than the ones in the cyan circle. When the risk feature ${\pmb {h}}_{r}$ was combined for time prediction, it guided the model to calibrate time predictions such that the death rankings among patients could be better preserved. As a result, the time predictions inside the blue circle were increased, whereas the ones inside the cyan circle were decreased, as shown in Fig. 2b. Note that the final time prediction results are closer to the ground truth with more correct death rankings.

In addition, we conducted the experiment by averaging the risk feature ${\pmb {h}}_{r}$ and time feature ${\pmb {h}}_{t}$ rather than concatenating them. As shown in Table 4, the averaging resulted in a slight improvement on BRCA but degradation on BLCA and GBM compared with concatenation; thus, we adopted concatenation as the feature fusion strategy in our experiments.

Instead of pre-training the RP-GCN and using it as a frozen risk feature extractor, we also conducted an ablation study by jointly training the RP-GCN and TP-GCN. As can be seen from Table 4, jointly training these two networks leads to higher MAEs and lower C-index scores. We emphasize that RP-GCN is intended to provide more accurate sequential guidance for time prediction; thus, training two networks simultaneously will make RP-GCN optimized not only by the cox loss but also a set of cross-entropy losses, which is uncorrelated to ranking prediction.

We further visualized the position of nodes that remained after the last *SagPool*^41^ operation. Since the *SagPool*^41^ operation is based on self-attention mechanism, the remaining nodes can be naturally considered as a high-attention area of RP-GCN and TP-GCN. As can be observed from Fig. 4, RP-GCN and TP-GCN showed distinct differences in areas of high attention. In other words, the time feature and risk feature convey complementary information for more accurate predictions.

**Table 4:** Ablation studies for different training strategies.

Feature		Train		BLCA		BRCA		GBM	
Concat	Avg	RP-GCN	TP-GCN	MAE	C-index	MAE	C-index	MAE	C-index
	$\checkmark$		$\checkmark$	192.33	0.814	165.7	0.621	312	0.511
$\checkmark$		$\checkmark$	$\checkmark$	162.8	0.801	180.8	0.500	304.4	0.549
$\checkmark$			$\checkmark$	123.2	0.849	167.5	0.605	303.3	0.554

**Figure 3:**
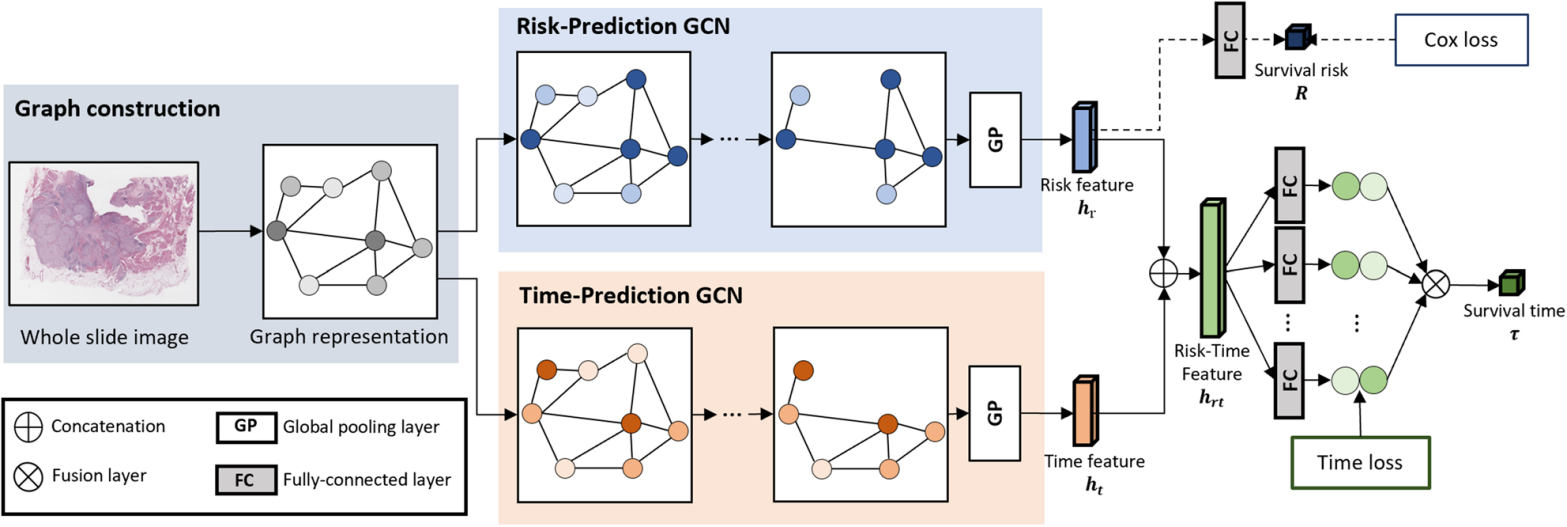
Overview of the overall workflow. It contains a graph construction, a survival risk prediction, and a survival time-prediction module. The graph is constructed using the tumor-like patches selected by our pre-trained tumor classifier. The survival risk-prediction GCN is pre-trained using the Cox model^21^ and frozen during time-prediction GCN training. The time-prediction GCN is trained by following the ordinal regression strategy^15^.

**Figure 4:**
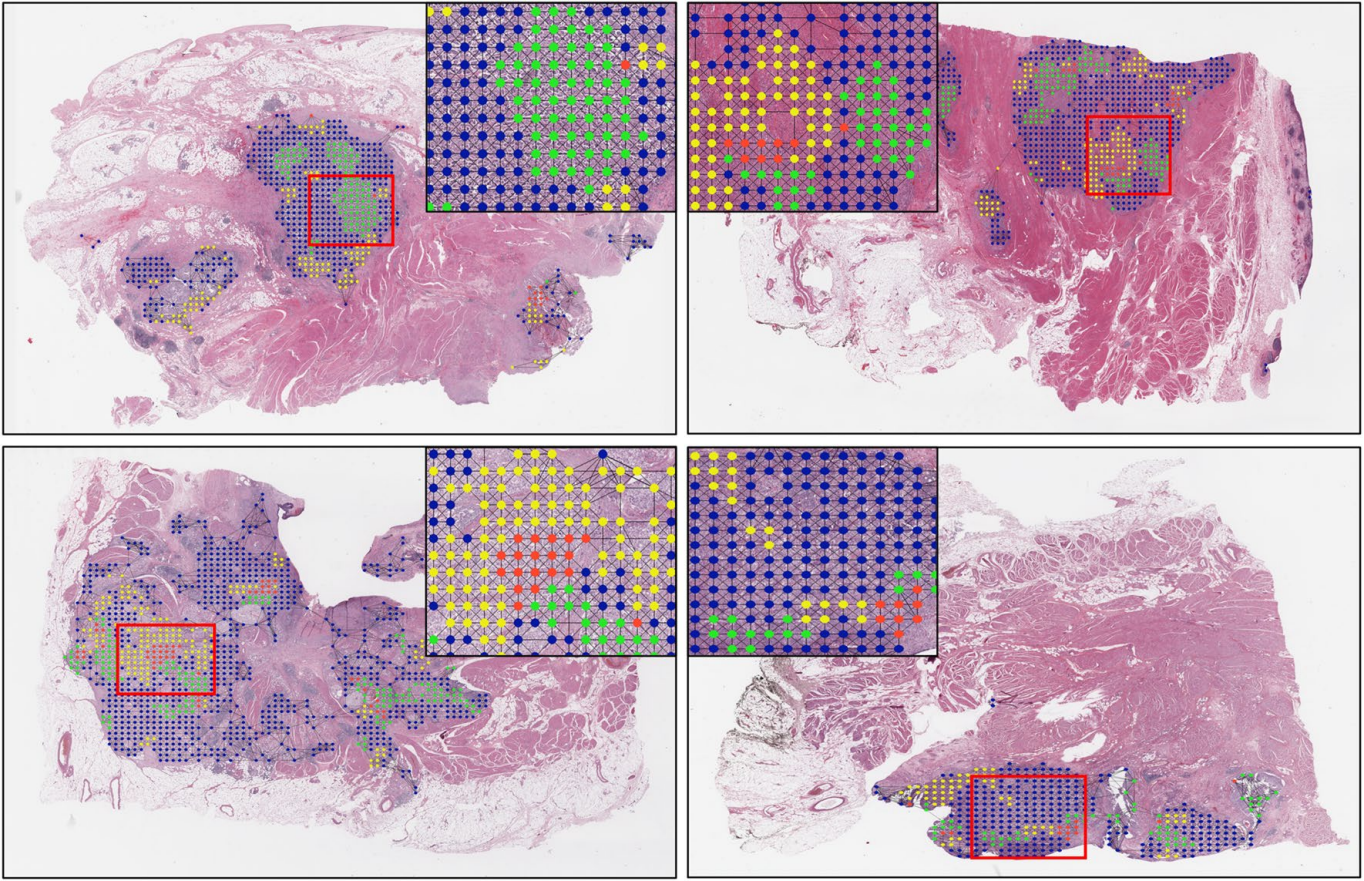
Visualization of highly attentive nodes for RP-GCN and TP-GCN. Blue indicates the nodes dropped from both networks after the last *SagPool* layer, yellow and green indicate the nodes remaining for RP-GCN and TP-GCN, respectively, and red indicates the overlapped nodes. The aspect ratio of images has been modified for better visualization.

## Conclusion and discussion

In this paper, we presented a novel approach for accurate survival time prediction that addresses this problem from two perspectives. First, the information available in a WSI needs to be aggregated globally without any intervention. Second, in addition to estimating the survival times of patients based on the characteristics of the patients themselves, the characteristics and survival times of other patients can also be used as comparison objects to estimate the survival times of target patients comprehensively. Considering these two perspectives, we proposed a two-branch GCN-based model that exploits the Cox model to capture the ranking relationships among patients in the form of the risk feature. The risk feature was then concatenated with the time feature to guide the model in making more precise time predictions with correct rankings. Experimental results on three public datasets demonstrated that the proposed risk-aware prediction method yielded more accurate survival time prediction results compared to its risk-agnostic version.

Several directions for future studies should be considered. First, we pre-trained the RP-GCN and fixed it during the training of the TP-GCN because our primary objective was a precise survival time prediction. For certain applications in which survival risk is more important, we expect that the proposed model can be trained in the opposite manner by using the time feature as a guide for risk prediction. Second, we used a pre-trained patch classifier to extract tumor patches for the graph construction. End-to-end training from the WSIs to survival time predictions is a challenging but promising direction for our future work. Last, our framework considered each WSI as an individual during training, and the final patient-level prediction was performed by taking the average of WSI-level predictions. We plan to apply an early fusion of WSI-level information to enable more precise patient-level predictions.

## Methods

### Problem formulation and motivation

In general, an instance of survival data can be represented as $\{{\pmb {x}}, \tau , \delta \}$, where ${\pmb {x}}$ is the feature vector of the patient and $\delta$ is the censoring status indicator, i.e., 1 for uncensored (death observed) data and 0 for censored data. $\tau$ is the last observation time for censored data or the survival time for uncensored data. The goal of survival time prediction is to predict $\tau$ by using ${\pmb {x}}$ and $\delta$.

One of the most widely used methods for survival prediction is the construction of the Cox proportional hazards model^21^. The hazard function, which assesses the relationship between the distribution of failure time (death time for survival prediction) and $\pmb {x}$^21^, is defined as $h(\tau |{\pmb {x}}) = h_0(\tau )exp({\pmb {\beta }}^T {\pmb {x}})$, where $h_0(\tau )$ is the baseline hazard function; and ${\pmb {\beta }}$ is a regression parameter vector. Katzman et al.^5^ first proposed a deep-learning-based method to directly predict the survival risk *R* (=${\pmb {\beta }}^T {\pmb {x}}$) using a fully connected network, demonstrating its superiority over the conventional regression-based methods. The survival risk *R* can be estimated by minimizing the negative log partial likelihood *l*(*R*), which is defined as follows:

**Figure 5:**
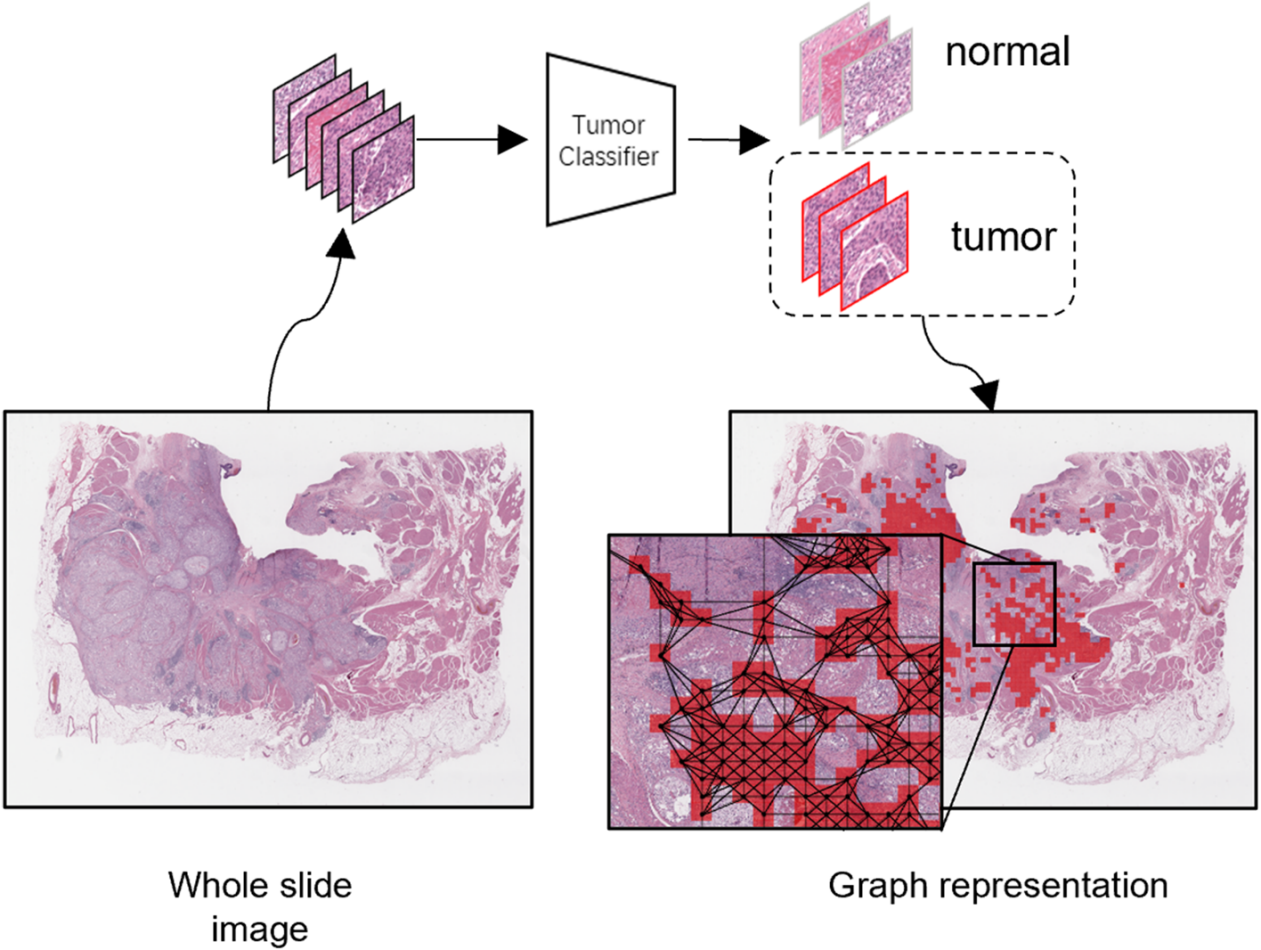
Illustration of the graph construction. We first remove the background of WSIs using the method in^42^. Then, the tissue regions are cropped into non-overlapping patches of size 512$\times$512 at the 20$\times$ magnification scale. Our pre-trained tumor classifier is used to select tumor-like patches, and the selected patches are connected by the *k*-NN algorithm in Euclidean space with a spatial distance threshold *d*. Features of each patch are extracted by an ImageNet^43^ pre-trained ResNet-18^33^.

4 $$\begin{aligned} l(R)=-\sum _{i=1}^M \delta _i \left( R_i -\log \sum _{j:\tau _j\ge \tau _i}\exp \left( R_j\right) \right) , \end{aligned}$$

where *M* is the number of samples, and the subscript *i* represents the data index. *l*(*R*) has been frequently used as the loss function for training survival risk prediction models^14,16–19,27–29^.

The Cox proportional hazards model includes the baseline hazard function, which is very challenging to estimate^15^. Therefore, an alternative method is to estimate the survival time $\tau$ directly from the feature vector ${\pmb {x}}$^15^. However, it does not consider that the risk orders among patients convey vital information^44^. Therefore, we propose to use the risk prediction features as guidance for the survival time prediction model such that the model can take advantage of the features capable of ranking between patients to predict more accurate results. We believe that in this manner, the results are closer to the ground truth compared to the one that does not use the risk prediction features.

### Proposal overview

The overall workflow of the proposed method is shown in Fig. 3. For a given WSI, we first apply a graph construction to generate the graph representation of the WSI, which will be described in “5.3” Graph construction section. Two GCNs are then utilized, namely the risk-prediction GCN (RP-GCN) and time-prediction GCN (TP-GCN), where we adopt the network architecture from^36^. While the former is pre-trained to predict the survival risk using the Cox model and frozen, the latter is trained by concatenating features from the former to guide survival time prediction. Specifically, given the concatenated risk-time feature vector, ordinal regression^15^ is performed with multiple fully connected layers to predict a series of binary outcomes, and then they are fused to the final survival time.

### Graph construction

Before constructing the graph, the background regions are removed using the method proposed in^42^. The remaining foreground tissue regions are cropped into non-overlapping patches of size $512\times 512$ (for the 20$\times$ magnification scale). To avoid the need for manual intervention by medical experts, a patch-level binary (normal/tumor) classifier is used to extract tumor-like patches from a WSI automatically. We adopt ResNet-18^33^ as the backbone for our classifier and train it using the Camelyon17 dataset^40^ which contains WSIs with pixel-level ROI annotations. The result is a set of tumor patches ${\pmb {\rho }=\{\rho |f(\rho )>0.5\}}$, where *f* is the tumor classifier that outputs the tumor probability of an input patch $\rho$. Then, the features of each patch in $\pmb {\rho }$ are extracted by an ImageNet^43^-pre-trained ResNet-18^33^, and they are treated as vertices in a graph, and edges between vertices are established by the *k*-nearest neighbors algorithm using the Euclidean distance between patch center positions with a spatial distance threshold *d* because the patches closer each other are more likely to interact^11^. The constructed graph can be represented as $G = (V,E)$, where *V* is a set of vertices, and *E* is the set of edges in the graph. The graph construction procedure is illustrated in Fig. 5.

5 $$\begin{aligned} e_{ij}=\left\{ \begin{matrix} 1, \mathrm {if~}j\in ~knn(v_i)~\mathrm{{and}}~D(v_i,v_j)\le ~d,\\ 0, \textrm{otherwise}, \\ \end{matrix} \right. \end{aligned}$$

where $e_{ij}\in E$ indicates the edge between two vertices $v_i$ and $v_j$, *D*(, ) the Euclidean distance between the center positions of two patches, and $knn(v_i)$ indicates the k-nearest neighbors of $v_i$.

**Figure 6:**
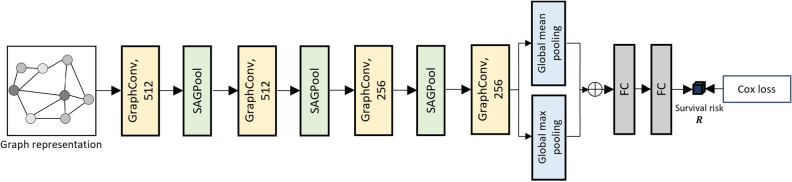
Illustration of RP-GCN. It contains four graph convolution layers and three self-attention graph pooling^41^ layers, followed by global max and mean pooling layers. Two fully connected layers are further applied for the survival risk estimation using the Cox model^21^. Note that these two fully connected layers are detached after the RP-GCN training is finished.

### Risk-prediction GCN

The generated graph *G* is first used for the RP-GCN shown in Fig. 6, which contains three GCN blocks and a single GCN layer, followed by a global max pooling, a global average pooling, and two fully connected layers. A GCN block can be expressed as follows:

6 $$\begin{aligned} \begin{array}{l} H^{(l+1)} = \sigma \left( SagPool\left( {\mathscr {G}}\left( H^{(l)}\right) \right) \right) , \\ {\mathscr {G}}\left( H^{(l)}\right) ={D}^{-\frac{1}{2}}{\tilde{A}}{D}^{-\frac{1}{2}}H^{(l)}W^{(l)}, \end{array} \end{aligned}$$

where ${\mathscr {G}}$ represents the graph convolution operation^36^ for one GCN layer, in which *A* is the adjacency matrix, and ${\tilde{A}}=A+I$ is the adjacency matrix including self-loops. *D* is the degree matrix, where its diagonal entry is given as ${D}_{ii}=\sum {_j}{\tilde{A}}_{ij}$, $H^{(l)}$ is the feature matrix at the *l*-th layer with $H^{(0)}=V$, and $W^{(l)}$ is the trainable parameters for layer *l*. $\sigma$ is an activation function, and *SagPool* represents the self-attention graph pooling^41^ with a pooling ratio *r*.

After graph convolutions, the risk feature vector ${\pmb {h}}_{r}$ is obtained by applying global max pooling and average pooling. We train the RP-GCN to predict survival risk *R* using (4) from ${\pmb {h}}_{r}$ via two fully connected layers, which are detached after the training of the RP-GCN is completed. The pre-trained RP-GCN is fixed and used as a risk feature extractor during the training of the TP-GCN to help predict more precise time predictions with correct rankings.

### Time-prediction model

As illustrated in Fig. 3, our overall survival time prediction model consists of the RP-GCN, TP-GCN, and multiple fully connected layers. Note that the same graph structure *G* and GCN architecture are used for both the TP-GCN and the RP-GCN. The risk feature vector ${\pmb {h}}_{r}$ extracted by the pre-trained RP-GCN is concatenated with the time feature vector ${\pmb {h}}_{t}$ extracted from the TP-GCN, resulting in the risk-time feature vector ${\pmb {h}}_{rt}$.

Following the ordinal regression framework^15^, we divide the range of survival time into *N* intervals, i.e., $\left\{ T_0,T_1,...,T_N\right\}$ with $T_0<T_1<...<T_N$. The intervals are empirically chosen to evenly distribute patients’ survival time in the training database. We then define a binary label for each patient as follows:

7 $$\begin{aligned} b_{n}=\left\{ \begin{aligned} 1, \textrm{if}~\tau >T_{n}, \\ 0, \textrm{otherwise}, \\ \end{aligned} \right. \end{aligned}$$

where $\tau$ is the survival time (or the last observation time for the censored data), and *n* is the index of the time interval. In this manner, the survival time prediction is transformed into an ordinal regression problem aiming to solve *N* binary classification where the *n*-th classifier predicts whether $\tau$ is greater than $T_{n}$. We adopt the censoring-aware loss^15^ so that both uncensored and censored data can be involved during training. The loss function is given as:

8 $$\begin{aligned} \begin{aligned} L(\pmb {o},\pmb {B},\pmb {\delta })=&-\sum _{n=1}^{N}[\delta _n+(1-\delta _n)b_{n}]\times [b_{n}\log (o_{n})+(1-b_{n})\log (1-o_{n})], \end{aligned} \end{aligned}$$

where $\pmb {B} = \left\{ b_1, b_2, \cdot \cdot \cdot , b_N \right\}$ and $\pmb {\delta } = \left\{ \delta _1, \delta _2, \cdot \cdot \cdot , \delta _N \right\}$. The output of the *n*-th classifier is denoted as $o_n$, and $\pmb {o} = \left\{ o_1, o_2, \cdot \cdot \cdot , o_N \right\}$. This means that for uncensored training samples, the cross-entropy is calculated for all $b_n$. For censored training samples, however, the last observation time is still valuable for training. Therefore, we ignore the intervals where $b_n=0$ and the loss is measured only when $b_n=1$.

In the testing stage, the final survival prediction time ${\tilde{\tau }}$ is calculated as:

9 $$\begin{aligned} {\tilde{\tau }}=\frac{{T_{\sum \limits _n{{\mathbb {I}}_{o_n}}}}+{T_{\sum \limits _n{{\mathbb {I}}_{o_{n}}+1}}}}{2}, \end{aligned}$$

where ${\mathbb {I}}_{o_n} = 1$ if $o_n \ge 0.5$, and ${\mathbb {I}}_{o_n} = 0$ otherwise.

## Data Availability

The datasets generated during and/or analysed during the current study are available from the corresponding author on reasonable request.

## Code availability

The code is available to editors and reviewers upon request. The code will be publicly available at https://github.com/ZXXu96/RAST upon acceptance.
